# Rosin Surfactant QRMAE Can Be Utilized as an Amorphous Aggregate Inducer: A Case Study of Mammalian Serum Albumin

**DOI:** 10.1371/journal.pone.0139027

**Published:** 2015-09-29

**Authors:** Mohd Ishtikhar, Tajjali Ilm Chandel, Aamir Ahmad, Mohd Sajid Ali, Hamad A. Al-lohadan, Ayman M. Atta, Rizwan Hasan Khan

**Affiliations:** 1 Protein Biophysics Laboratory, Interdisciplinary Biotechnology Unit, Aligarh Muslim University, Aligarh – 202002, India; 2 Karmanos Cancer Institute, Wayne State University, School of Medicine, 707 HWCRC 4100 John R. St., Detroit, MI 48201, United States of America; 3 Surfactant Research Chair, Department of Chemistry, College of Science, King Saud University PO Box-2455, Riyadh–11541, Saudi Arabia; Russian Academy of Sciences, Institute for Biological Instrumentation, RUSSIAN FEDERATION

## Abstract

Quaternary amine of diethylaminoethyl rosin ester (QRMAE), chemically synthesized biocompatible rosin based cationic surfactant, has various biological applications including its use as a food product additive. In this study, we examined the amorphous aggregation behavior of mammalian serum albumins at pH 7.5, i.e., two units above their isoelectric points (pI ~5.5), and the roles played by positive charge and hydrophobicity of exogenously added rosin surfactant QRMAE. The study was carried out on five mammalian serum albumins, using various spectroscopic methods, dye binding assay, circular dichroism and electron microscopy. The thermodynamics of the binding of mammalian serum albumins to cationic rosin modified surfactant were established using isothermal titration calorimetry (ITC). It was observed that a suitable molar ratio of protein to QRMAE surfactant enthusiastically induces amorphous aggregate formation at a pH above two units of pI. Rosin surfactant QRMAE-albumins interactions revealed a unique interplay between the initial electrostatic and the subsequent hydrophobic interactions that play an important role towards the formation of hydrophobic interactions-driven amorphous aggregate. Amorphous aggregation of proteins is associated with varying diseases, from the formation of protein wine haze to the expansion of the eye lenses in cataract, during the expression and purification of recombinant proteins. This study can be used for the design of novel biomolecules or drugs with the ability to neutralize factor(s) responsible for the aggregate formation, in addition to various other industrial applications.

## Introduction

In recent years, protein aggregation-related diseases have emerged as the topics of interest in the field of protein engineering. Protein unfolding occurs due to conformational alteration under harsh conditions like high or low pH, higher temperature, different types of surfactants and cosolvents in which protein losses its native and active conformation, and gets unfolded or aggregated [1–5]. Therefore, protein requires it’s correctly folded conformation for the biological activity [6]. Conformational alterations are generally recognized to play a pivotal role in the aggregation process leading to the exposure of hydrophobic groups of protein that are normally present in buried regions [7]. These hydrophobic groups may initiate the formation of new intermolecular interactions. The conformational alterations may initiate different aggregation pathways which result in the formation of different supramolecular aggregated structures such as amorphous aggregates or amyloid fibrils [8].

Generally, in the aggregation process, aggregate size may be affected by pH which initially determines the structure and the net charge present on the protein surface, and also determines the different kinds of interactions which are involved [3]. In these circumstances, the exploration of the pH dependent aggregation process of a suitable protein can help to elucidate the mechanisms of aggregation. The amorphous aggregation of proteins is associated with various phenomena, starting from the formation of protein haze white wine [9] to the expansion of eye lens in cataract [10] and during the expression and purification of recombinant proteins precipitation [8]. The eye lens crystalline protein is destabilized due to life-long radiative and oxidative stress-mediated unfolding process leading to aggregation. These aggregates act as precursors of the cataract formation in the aged people [11]. Despite amorphous protein aggregation being such a significant problem, it has received little attention in past decades and is often over shadowed by studies of protein aggregation that involves linear structured amyloid fibrils associated with neurodegenerative disorders such as Alzheimer's, Parkinson's, Huntington's, Senile systematic amyloidosis, type II diabetes and many others [12–14]. Moreover, unlike the highly structured linear amyloid fibrils, the amorphous aggregation is a disordered three-dimensional process where monomer can adjoin from every direction. Hence, the increase in aggregate size and area is directly proportional to the concentration of available monomers [8].

It is known that a majority of the proteins may acquire entirely folded and functional conformation under *in vivo* conditions, but some proteins undergo misfolding due to various reasons [15]. It is still unclear whether amyloid or amorphous aggregate formation is commenced by native state of proteins or totally/partially unfolded states [16, 17]. The unfolding and aggregation of serum albumins has been widely studied *in vivo* as well as *in vitro* by different research groups [12, 18–21]. Various strategies have been proposed to achieve stabilized form of proteins, including chemical modification, protein engineering, use of surfactants, rosin modified compounds and polyhydroxy compounds [3, 22, 23].

Serum albumins play a vital role in the transportation and distribution of exogenous and endogenous ligands via blood stream [24, 25]. They also help to maintain the osmotic pressure and physiological pH of the blood [25, 26]. Human serum albumin (HSA), bovine serum albumin (BSA), porcine serum albumin (PSA), rabbit serum albumin (RSA) and sheep serum albumin (SSA) are globular proteins, consisting of a single polypeptide chain, and belong to α-class of proteins that have average molecular weight of approximately 66.5 kDa [26]. They have three structurally similar α-helical domains (I, II, III). Each domain is divided into sub-domains A and B, which contain six and four α-helices, respectively [27]. Out of six, two sub-domains (IIA and IIIA) have high binding affinity sites for small heterocyclic or aromatic compounds [25, 28, 29].

In present study we have used quaternary amine of diethylaminoethyl rosin ester (QRMAE), a chemically synthesized biocompatible rosin based cationic surfactant of rosin (abietic acid) is one of the essential natural renewable assets with excellent solubility that is cheaply available in the market [30]. QRMAE is a water soluble rosin surfactant that was prepared from condensed products of rosin formaldehyde derivatives [31], and etherified with different molecular weights of polyethylene-glycols to prepare nonionic and cationic polymeric surfactant as reported previously [32, 33]. Rosin derivatives have bulky hydrophenanthrene group, are hydrocarbon rich and used as food additives because of their hydrophobicity and significantly altered thermal property of integrated polymers [34].

In present work, we have studied how QRMAE induces amorphous aggregation of a number of mammalian serum proteins when subjected at pH two units above their isoelectric point (pI). It is interesting to investigate the interaction of surfactant with mammalian serum albumins in an attempt to understand the function of such types of interactions in the protein aggregation process. Utilizing biophysical methods, we confirmed that aggregate formation principally initiated by electrostatic interactions that was further governed by hydrophobic interactions [3, 7]. However, the role of QRMAE (similar to fatty acids) in the field of aggregation is still very complex and unpersuasive. In this work, we propose how QRMAE can possibly induce the amorphous aggregation of mammalian serum albumin above two units of pI and we also list the factors that trigger the formation of amorphous aggregate.

## Materials and Methods

Human serum albumin (A1887), bovine serum albumin (A7030), porcine serum albumin (A1830), rabbit serum albumin (A0764), sheep serum albumin (A3264), thioflavin T (ThT) and 1-anilino-8-naphthalene sulfonate (ANS) were procured from Sigma Aldrich (USA), whereas sodium di-hydrogen orthophosphate, di-sodium hydrogen orthophosphate, sodium acetate and glacial acetic acid buffer components were procured from Qualigens, India. Double distilled water was used throughout the study. QRMAE (Mol. wt. 697) was synthesized as described in the previous work [35, 36].

### Protein concentration determination and sample preparation

A stock solution of mammalian serum albumin was made in 20 mM sodium phosphate buffer of pH 7.5 and protein concentration was determined spectrophotometrically at 280 nm by using $E_{1cm}^{1\%}$ of 5.3 (HSA), 6.7 (PSA), 6.72 (SSA) and 43834 M^-1^cm^-1^ (BSA) 43385 M^-1^cm^-1^ (RSA) on JASCO V-660 spectrophotometer [3]. A buffer solution of 20 mM sodiam-glacial acetic acid was prepared for pH 3.5. Buffers used in these experiments were filtered through a 0.45 μm Millipore Millex-HV PVDF filter and pH was measured using Mettler-Toledo pH meter (model S20). All reagents used in the study are of analytical grade. Rosin surfactant QRMAE, synthesized as described in the previous work [32], was directly dissolved in the respective buffers. The rosin surfactant compound QRMAE was of high purity as it was re-crystallized many times and characterized by FTIR and NMR spectra, along with surface tension measurements which suggest that the QRMAE was free from any impurity [32, 36, 37]. The chemical structures and scheme of QRMAE surfactant synthesis are illustrated as in S1 Fig.

### Turbidity measurements

Turbidity measurements were performed on a JASCO V-660 double beam UV-Visible spectrophotometer in a cuvette of 1 cm path length. The turbidity of control mammalian serum albumins (5 μM), as well as in the presence of 0.25 mM surfactants, was determined by monitoring the changes in absorbance at 350 nm. All the samples were incubated at 65°C before the respective sample time measurements.

### Rayleigh light scattering (RLS) measurements

Rayleigh light scattering measurements were taken on a Shimadzu (RF-5301PC) fluorescence spectrophotometer at room temperature in a cuvette of 1 cm path length. The samples were excited at 350 nm and spectra were recorded from 300 to 400 nm, therefore RLS values were noted at 350 nm. Both excitation and emission slit width were set at 1.5 nm. The protein sample without surfactant served as control. All the samples were incubated at 65°C prior to measurements.

### ANS binding assay

A fresh stock solution of ANS was prepared in double distilled water and filtered with 0.2 micron Millipore filter. The concentration of ANS was measured using molar extinction coefficient ε_M_ = 5000 M^-1^cm^-1^ at 350 nm [38]. Post incubation, samples were supplemented with ANS solution in the ratio of 1:20 and further incubated for 30 minutes in the dark. ANS emission spectra were recorded from 390 to 600 nm by using an excitation wavelength of 380 nm. The excitation and emission slit widths were set at 3 and 5 nm, respectively. Fluorescence measurements were made by using a Shimadzu (RF-5301PC) fluorescence spectrophotometer.

### ThT binding assay

A stock solution of ThT was prepared in double distilled water and filtered with 0.2 micron Millipore filter. The concentration of ThT was measured using molar extinction coefficient ε_M_ = 36000 M^-1^cm^-1^ at 412 nm [3]. The protein samples, in the absence or in the presence of surfactants, were incubated at 65°C. Post incubation, samples were supplemented with ThT solution in the ratio of 1:05, and further incubated for 30 minutes in the dark. The ThT was excited at 440 nm and spectra were recorded from 450 to 600 nm. The excitation and emission slit widths were set at 5 and 10 nm, respectively. Fluorescence measurements were made by using a Shimadzu (RF-5301PC) fluorescence spectrophotometer.

### Far and Near-UV CD measurements

The circular dichroism measurements were performed on a JASCO spectropolarimeter (J-815) with a thermostatically controlled cell holder attached to a peltier with multitech water circulator. The experiments were carried out at room temperature, far and near spectra were scanned in the range of 200–250 nm and 250–320 nm in a cuvette of 1 or 10 mm pathlength, respectively. Each spectrum was an average of three scans. The spectra were smoothed by the Savitzky-Golay method with 15 convolution width. The results may be expressed as mean residual ellipticity (MRE) in deg cm^2^ dmol^-1^ which is defined as:

$$\text{MRE}=\frac{\theta_{obs}(m\text{deg})}{10\times n\times C\times l}$$ (1)

Where θ_obs_ is the observed ellipticity in degrees, *C* is the molar fraction, *n* is the number of amino acid residues and *l* is the length of the light path in centimeter.

Helical content was calculated from the MRE values at 222 nm using the following equation as described by Chen *et al*.[39], which was further supported by the K2D software:

$$\%\alpha \;-\text{helix }=\;\left(\frac{{\text{MRE}}_{222\text{ nm}}\;-2,340}{30,300}\right)\times 100$$ (2)

### Fourier transforms infrared (FTIR) measurements

FTIR spectra were recorded at 25°C using Perkin Elmer spectrum two FTIR spectrometer equipped with a zinc selenide (ZnSe) attenuated total reflectance (ATR) accessory, a deuterated triglycine sulfate (DTGS) detector and KBr beam splitter. Aliquots of 20 μL from each protein sample solution (5 mg/ml) in the absence and presence of rosin surfactant (3.75 mM) were placed between CaF_2_ windows separated by a 50 mm polyethylene terephthalate spacer. Each spectrum represents the average of 100 scans recorded in the region from 1250 to 1850 cm^-1^. Blank spectra were substracted from corresponding spectra.

### Isothermal titration calorimetric (ITC) measurement

The thermodynamic parameter of the binding and aggregation of albumins with QRMAE at 65°C were measured by using VP-ITC titration microcalorimeter (MicroCal Inc., Northampton, MA). Prior to the titration experiment, all samples were degassed on a Thermovac. The sample and reference cell of the calorimeter were loaded with serum albumin solution (15 μM) and 20 mM phosphate buffer (pH 7.5) near to physiological pH, respectively. Multiple injections of 10 μL of QRMAE solution of 2.5 mM concentration were made into the sample cell containing mammalian serum albumins. Each injection was made over 20 s with an interval of 180 s between successive injections. The reference power and stirring speed were set at 16 μcal s^-1^ and 307 rpm [29], respectively. Heats of dilution for the ligands were determined in control experiments, and these were subtracted from the integrated data before curve fitting.

### Differential scanning calorimetry (DSC) measurement

Thermal denaturation experiments were conducted on a VP-DSC microcalorimeter (MicroCal, Northampton, MA). The DSC scans were run between 20°C and 90°C at a rate of 1°C min^-1^. The experiments were performed using 15 μM concentration of serum albumin. The respective reference scans were run under identical DSC set up conditions and subtracted from each sample scan. The heat capacity curves, midpoint temperature (*T*_m_), calorimetric enthalpy (*ΔH*_cal_) and van’t Hoff enthalpy (*ΔH*_vH_) were analyzed using Origin 7.0 software.

### Scanning electron microscopic (SEM) measurements

The micro-architecture and morphology of serum albumin aggregate was resolved by using SEM. Air dried samples were placed on a gold-palladium grid which was attached to carbon tape, facilitating the sticking of the sample on the grid. The samples were imaged by using JSM-6510LV (JEOL Japan) scanning electron microscope at the voltage of 10 kV.

### Fluorescence microscopic (FM) measurements

To further characterize the morphological structure of aggregated serum albumins, we used the specific amorphous aggregate binding dye ANS, which was visualized by fluorescence microscopy. Serum albumin aggregates obtained after incubation time points were incubated with 100 μM of ANS dye for 30 minutes at room temperature and then transferred to a glass slide. Now, the samples were imaged using fluorescence microscopy (Axio, HBU 50/AC; Zeiss, Gottingen, Germany). The images were acquired using a Carl Zeiss Imager M2 microscope (20x objective magnifications) equipped with AxioCam digital camera. The microscope is controlled by ZEN2010 image processing software. For precise imaging, Z-stacking was done in the FITC channel.

## Results

### Turbidity measurement

**Fig 1:**
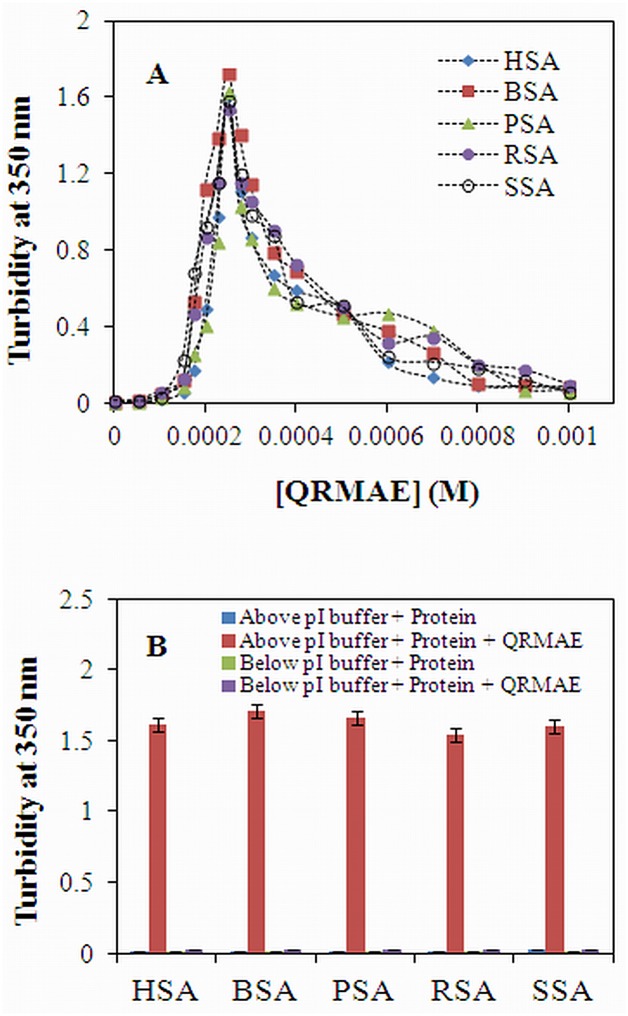
(A) Turbidity of the mammalian serum albumins samples at 350 nm were obtained in the absence and presence of varying concentrations of QRMAE to find out the maximum aggregation points at which surfactant concentration maximum aggregate was formed. (B) Turbidity measurement of protein samples in the absence and presence of rosin surfactant QRMAE at pH below and above two unit of their pI. Albumin concentration was fixed 5 μM in all conditions. The experiment was performed after 60 min incubation at 65°C.

The effects of QRMAE on aggregation were examined via turbidity measurements at 350 nm with respect to QRMAE concentration (0–1000 μM). The turbidity of proteins was monitored at 350 nm in order to determine the protein aggregation process. The absorbance at 350 nm by a double beam spectrophotometer is an effective method to detect the aggregation in proteins. Thus, the enhanced turbidity of the protein in the presence of QRMAE at pH two units above (pH 7.5) of proteins pI after 1 h incubation at temperature 65°C, gives information about the aggregate formation (Fig 1A) and the maximum turbidity were determined at 250 μM concentration of QRMAE surfactant. The turbidities of all five albumins were studied in the absence and presence of the rosin surfactant at two different pH values, i.e. two units above (pH 7.5) and two units below (pH 3.5) of proteins pI. But the enhanced absorbance at 350 nm was observed in the protein sample which was incubated with QRMAE surfactant at pH value above two units of its pI. Proteins incubated with QRMAE below its pI as well as proteins without QRMAE, below and above two units of its pI did not show any significant absorbance due to the absence of aggregation process (Fig 1B).

### Rayleigh light scattering (RLS) measurements

**Fig 2:**
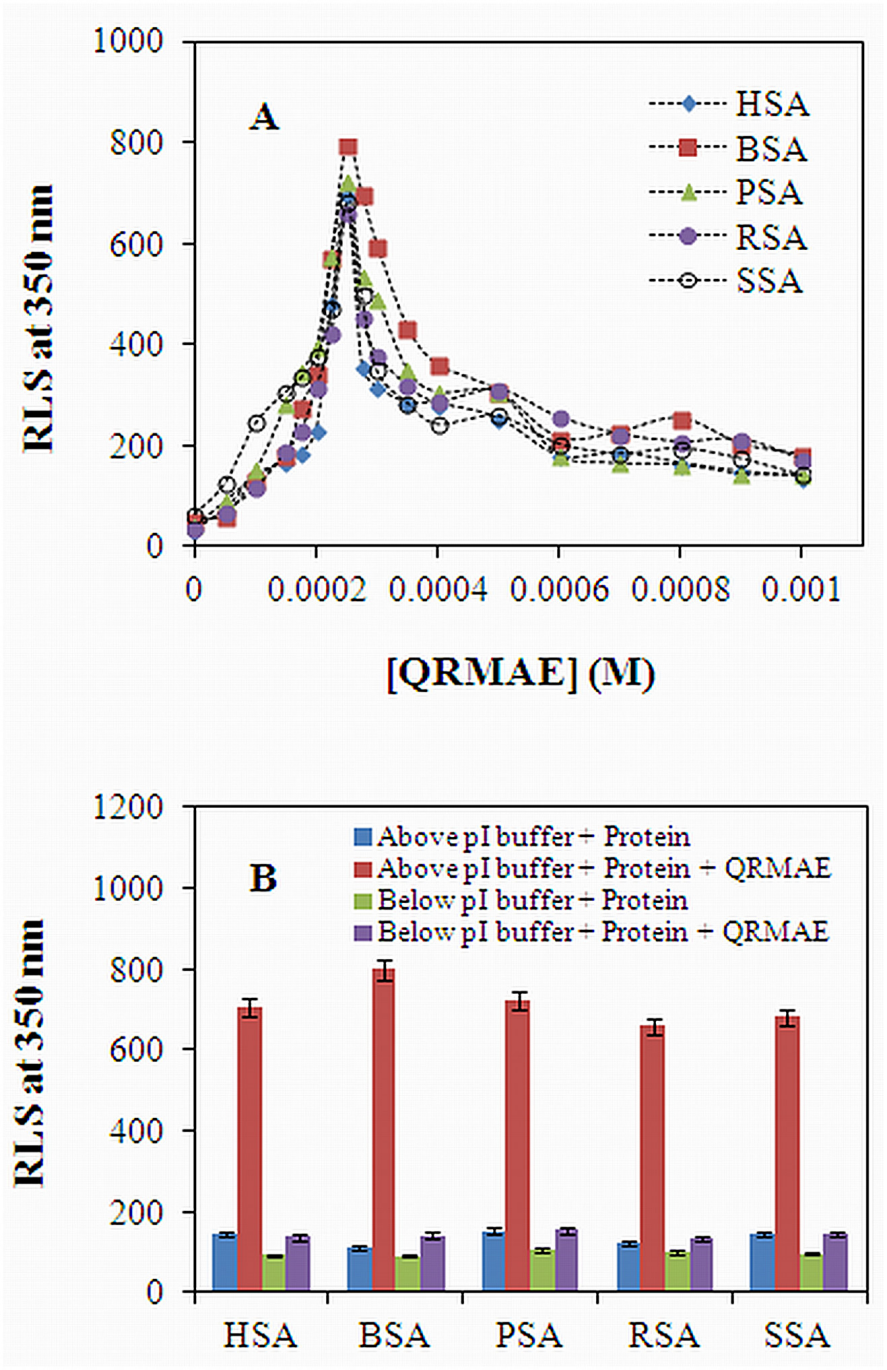
(A) RLS measurements of the mammalian serum albumins samples at 350 nm were obtained in the absence and presence of varying concentrations of QRMAE to find out the maximum aggregation points at which surfactant concentration maximum aggregate was formed. (B) RLS measurement of protein samples in the absence and presence of rosin surfactant QRMAE at pH below and above two unit of their pI. Albumin concentration was fixed 5 μM in all conditions. The experiment was performed after 60 min incubation at 65°C.

The aggregation propensity of QRMAE at different concentrations was once again analyzed by RLS measurements at 350 nm with the all five mammalian albumins in the absence and presence of rosin surfactant at pH two unit above and below of pI. This technique is highly sensitive in determining the aggregation or turbidity of proteins. The increase in fluorescence intensity at 350 nm of protein samples incubated with QRMAE at pH above two units of its pI revealed the extent of scattering of light in the protein. We found that the maximum intensity was revealed at 250 μM concentration of QRMAE, only at pH above two unit of pI (Fig 2A). This information showed an excellent uniformity of the result obtained by the turbidity experiments. Furthermore, at that particular concentration of QRMAE, surfactant was incubated with all five types of albumins at two particular pH values i.e. two units above and below its pI. A noteworthy increase in fluorescence intensity (approximately several fold) was observed when proteins were incubated with QRMAE in buffer above two units of its pI at temperature 65°C, as compared to control samples (Fig 2B), The values are shown in Table 1. The turbidity measurement was highly supported by RLS results. Maximum absorption obtained by turbidity measurement and maximum fluorescence intensity shown by RLS analysis was found to be at the same concentration of the surfactant (Figs 1A and 2A). Thus, this ensures that the formation of aggregates occurs in all the five serum albumin samples at particular surfactant concentration and pH value.

**Table 1:** Spectroscopic property of mammalian serum albumins under different experimental conditions, in the absence and presence of rosin modified surfactant after 60 min incubation at 65°C.

pH	Systems	% α-Helicity	% β-Sheet	% Random Coil	ANS At 480 nm	ThT At 485 nm	RLS At 350 nm	Turbidity At 350 nm
7.5	HSA	57.80	08.67	33.53	191.06	41.76	145	0.0117
7.5	BSA	57.18	09.56	33.26	205.35	42.41	109	0.0138
7.5	PSA	60.61	10.24	29.15	165.88	32.45	153	0.0122
7.5	RSA	58.16	09.12	32.72	176.92	43.36	122	0.0132
7.5	SSA	62.85	09.83	27.32	176.26	35.29	144	0.0116
7.5	HSA-QRMAE	02.09	18.34	79.57	593.36	63.04	706	1.6174
7.5	BSA-QRMAE	01.57	20.19	78.24	642.05	57.97	799	1.7110
7.5	PSA-QRMAE	01.07	17.35	81.58	555.23	68.83	721	1.6583
7.5	RSA-QRMAE	06.39	18.59	75.02	515.45	56.46	660	1.5412
7.5	SSA-QRMAE	07.32	17.98	74.70	566.75	57.98	681	1.5978
3.5	HSA	51.91	11.29	36.80	142.97	21.79	92	0.0101
3.5	BSA	43.91	11.05	45.44	157.64	21.06	91	0.0103
3.5	PSA	54.65	10.92	34.43	128.08	19.22	108	0.0099
3.5	RSA	56.34	11.75	31.91	138.93	20.94	100	0.0107
3.5	SSA	61.96	12.28	25.76	140.81	20.99	97	0.0142
3.5	HSA-QRMAE	58.37	12.83	28.80	229.53	22.94	137	0.0258
3.5	BSA-QRMAE	50.85	13.62	35.53	224.56	20.29	142	0.0245
3.5	PSA-QRMAE	59.48	11.12	29.40	256.84	23.46	155	0.0237
3.5	RSA-QRMAE	55.54	12.32	32.14	254.22	21.44	134	0.0248
3.5	SSA-QRMAE	60.04	12.76	27.02	227.70	23.16	144	0.0269

### ANS binding study

**Fig 3:**
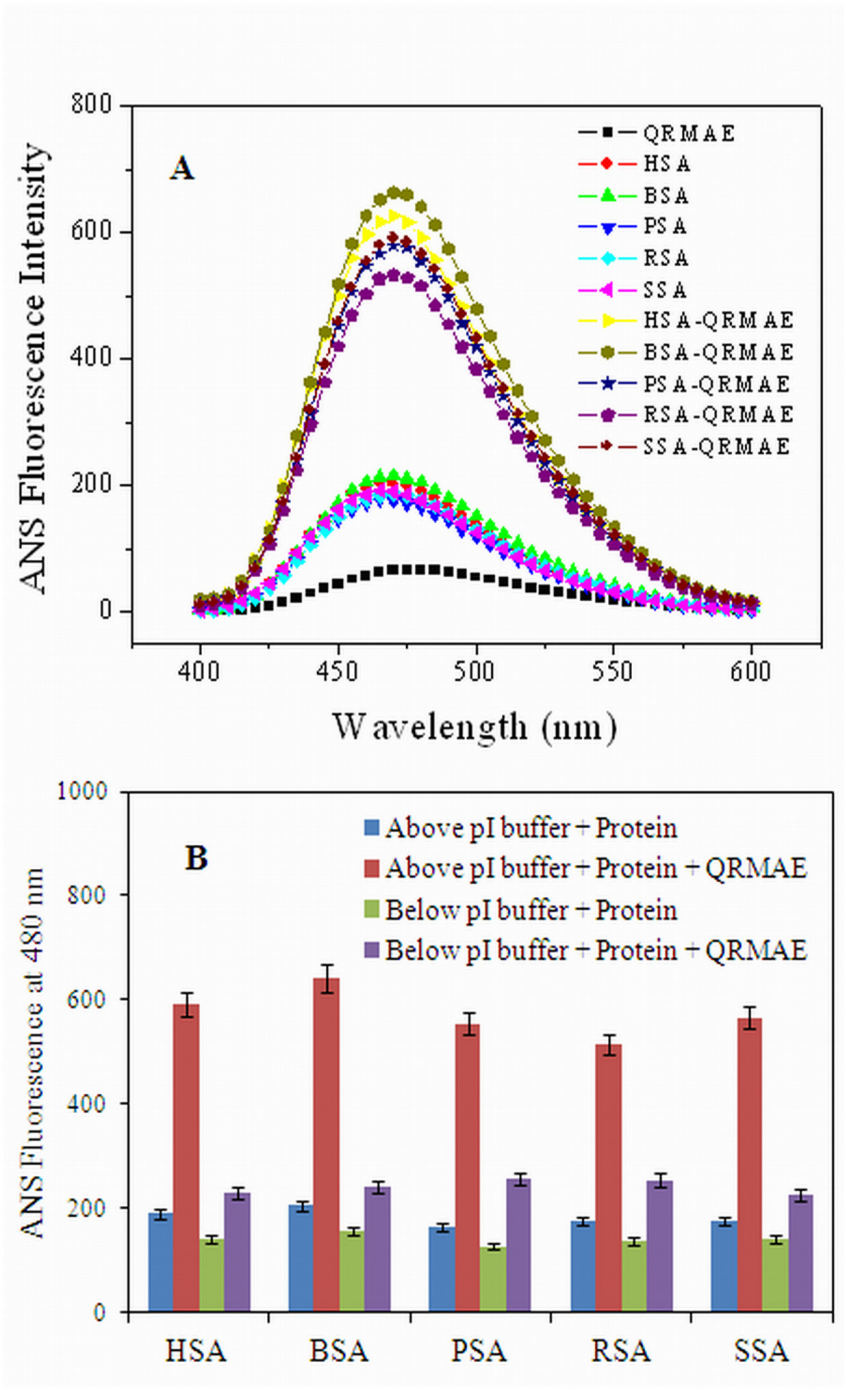
(A) ANS fluorescence spectra of mammalian serum albumins in the abscence and presence of QRMAE at pH above two unit of their pI and (B) ANS fluorescence intensity data of protein samples at 480 nm in the absence and presence of QRMAE at pH below and above two unit of their pI.

1-Anilino-8-napthalene sulfonic acid, is an ammonium salt widely used to characterize the nature of the protein aggregates. ANS binds with an amorphous aggregate and shows enhanced fluorescence intensity upon binding with aggregated particles in the protein samples. The protein samples were incubated at 65°C for an hour and then incorporated with ANS. Fluorescence spectra of QRMAE alone and the controls samples were found to be negligible at pH below two unit of pI, whereas, on the other hand, QRMAE-induced aggregation of mammalian serum albumins with ANS showed a characteristic high intensity spectra at pH above two unit of pI (Fig 3A), which suggested a binding of ANS with the amorphous or amyloid aggregates. ANS fluorescence intensities, measured at 480 nm after excitation at 380 nm, showed a negligible effect at pH below two units of their pI (Fig 3B). However, there is a significant difference in the fluorescence intensities between the protein samples above two units of its pI and the sample when incubated with QRMAE under pH below two unit of its pI. As shown in Fig 3 and Table 1; the fluorescence intensity obtained by the controls was very low, but when the protein was incubated with QRMAE above two units of its pI, there was an elevation in the intensities, approximately up to three-folds at 480 nm.

### ThT binding study

**Fig 4:**
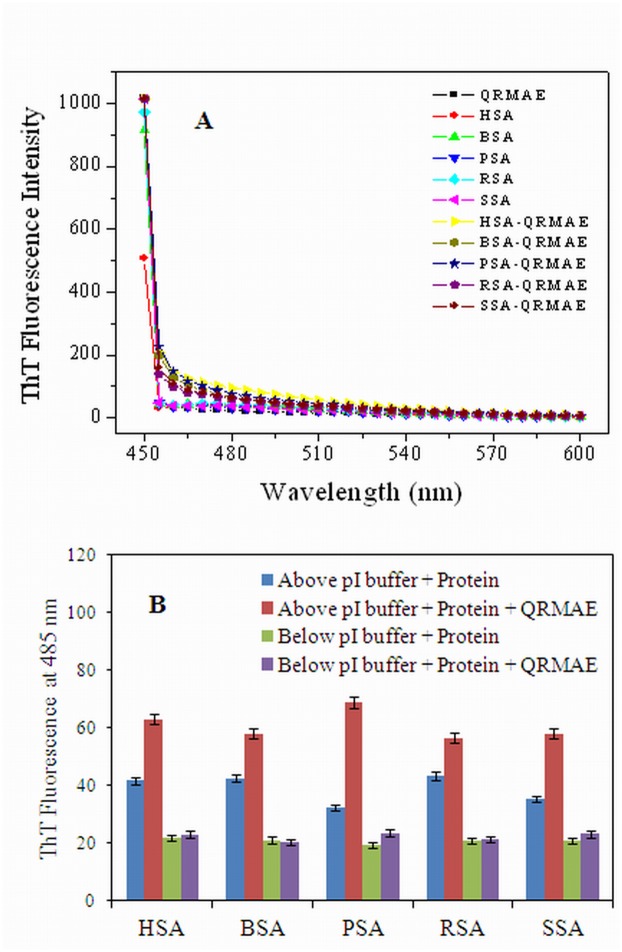
(A) ThT fluorescence spectra of mammalian serum albumins in the absence and presence of QRMAE at pH above two unit of their pI and (B) ThT fluorescence intensity data of protein samples at 485 nm in the absence and presence of QRMAE at pH below and above two unit of their pI.

Thioflavin T (ThT) is a benzothiozole dye, which is used to characterize amyloid fibrils by interacting with cross β-sheet structures of the proteins. ThT-binding to amyloid structures of protein exhibits a significant increase in the fluorescence intensities as compare to the other forms of aggregates. Therefore, ThT has insignificant intensity change at 485 nm towards amorphous or non-amyloid structures. Thus, ThT can distinguish different variants of aggregates in the protein sample incubated without or with QRMAE surfactant [40]. The albumin samples incubated at 65°C for an hour without or with QRMAE at pH below and above two units of their pI, showed negligible ThT fluorescence spectra change (Fig 4A). The insignificant ThT binding reveals that the aggregates present in the protein sample were amorphous, not an amyloid fibrils. This was further confirmed by Fig 4B, that showed the controls as well as albumins incubated with QRMAE had negligible ThT fluorescence intensities at 480 nm. Thus, the results of ThT binding confirmed that the thermally induced aggregates were amorphous in nature.

### Secondary and tertiary structure determination by circular dichroism (CD)

**Fig 5:**
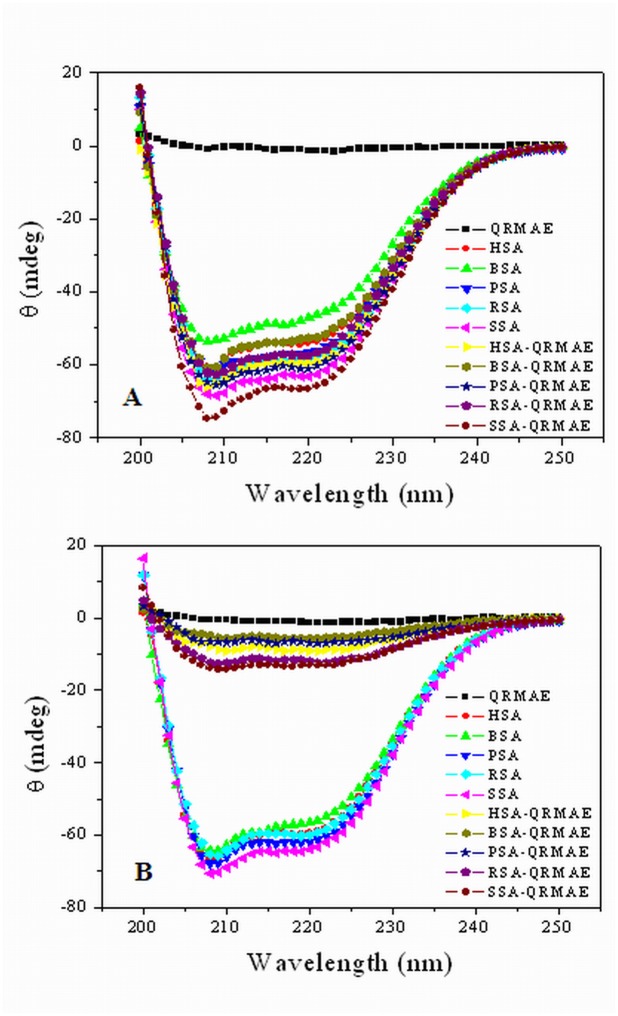
(A) Far-UV CD spectra of mammalian serum albumins at pH below two unit of proteins pI and (B) at pH above two unit of proteins pI in the absence and presence of rosin surfactant QRMAE. The experiment was performed after 60 min incubation at 65°C. [The presence of secondary structure, contents for α-class of protein was calculated by Chen et al. method that was further supported by K_2_D software analysis].

Far-UV CD measurements were used to determine the secondary structure change in the protein. The secondary structure consists of α-helix, β-sheet and random coil structure [29]. Two negative peaks of α-helix lies at 208 nm and 222 nm, one negative peak of β-sheet and one negative peak of random coil lies at 217 nm 197 nm, respectively [41]. Far and near-UV CD studies were used to determine the effect of QRMAE on the secondary and tertiary structure of proteins (albumins) at pH below and above two units of its pI. Proteins were incubated at 65°C for 1 h. It has been shown in Fig 5A that CD spectra of controls and QRMAE induced mammalian serum albumins at pH 3.5 (below two units of pI) exhibited two minima at 208 nm and 222 nm which indicated the formation of α-helical structures [42, 43]. On the other hand, at pH 7.5 (above two units of pI), a noticeable difference in the CD spectra of the albumins (Fig 5B) in the presence of rosin surfactant QRMAE was observed and the data obtained after % α-helisity calculation is listed in Table 1. A remarkable decrease in negative ellipticity of the QRMAE incubated proteins was observed, which indicated that the transformation of α-helical structure to β-sheet or random coil structure occurred due to aggregate formation. This confirmed that the cause of aggregation of albumins was QRMAE at pH two units above its pI and the protein structures still retained their α-helical and radom coil nature.

Alterations in tertiary structures of protein are best monitored by near-UV CD measurements. The actual shape and magnitude of protein spectra depends on the number of each type of aromatic amino acids and their mobility as well as the nature of their environments (H-bonding, polarisability and polar groups) and spatial position in the protein. The spectra in the range of 260–320 nm arise from the aromatic amino acids as well as the asymmetry of disulfide bridges [44, 45]. Each of the amino acids tends to have a characteristic wavelength profile. The native spectra of HSA at both pH values showed a flat band around 290 nm, which is specific for Trp residues (S2 Fig). It shows that the Trp residue is buried interior of the protein. Tyr and Phe have a characteristic peak between 275–282 and 255–270 nm [46]. However, on complexation of HSA with rosin surfactant at pH below and above pI, there was appearance of peaks at particular range of wavelength suggesting the effect of respective aromatic amino acid, At pH 7.5, in presence of surfactant, loss of the elliptisity was observed, as compared to HSA spectra in the absence of surfactant. This suggested a loss of tertiary structure of protein. Therefore, above changes in near-UV CD spectra suggested that at pH 7.5, in the presence of surfactant after incubation at 65°C for 1h, there is loss of tertiary structure of proteins, but at pH 3.5, in presence and absence of surfactant, no major changes are observed.

### Fourier transforms infrared (FTIR) analysis

**Fig 6:**
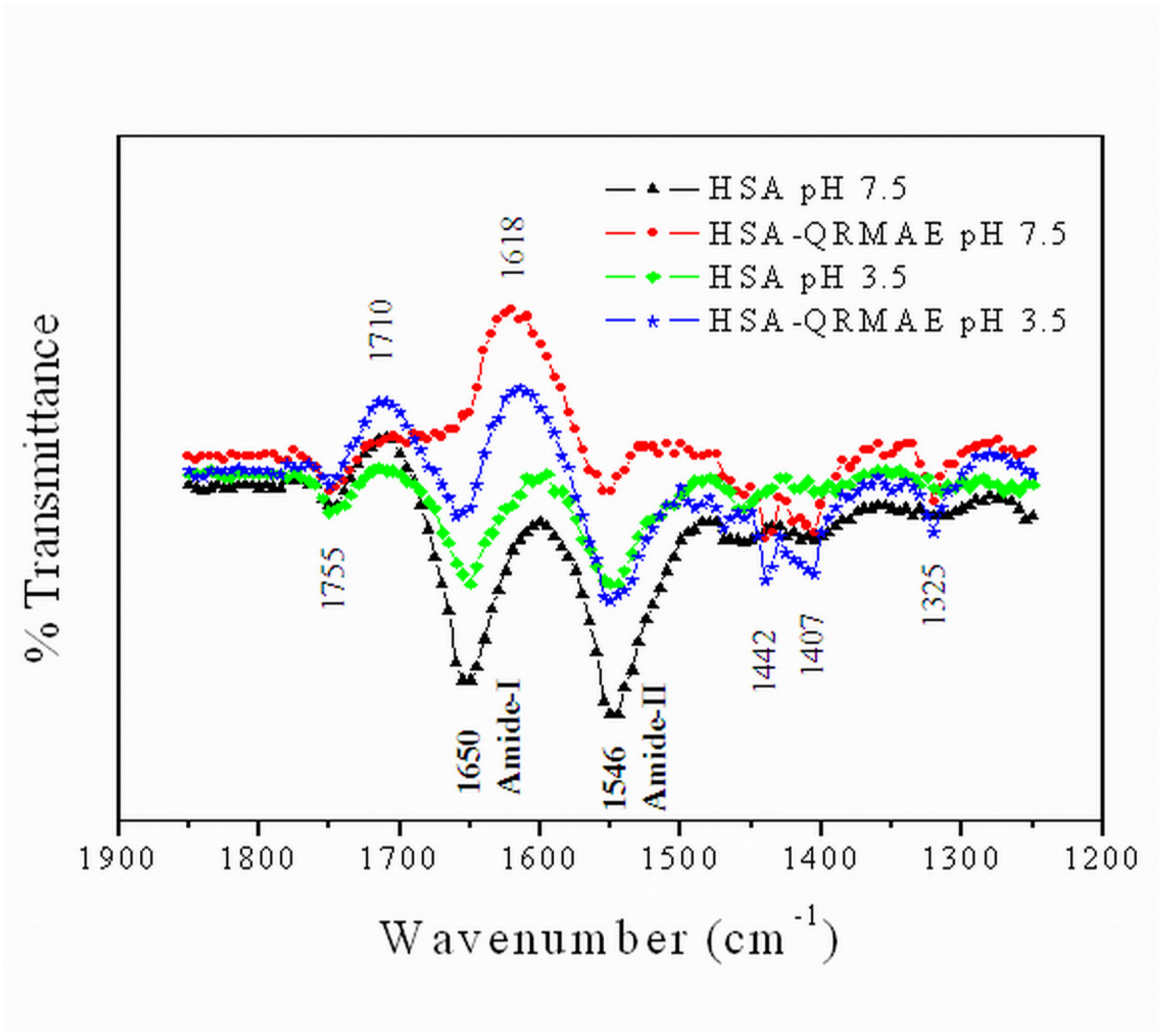
FT-IR spectra are showing Amide I and Amide II bands in the region of 1850–1250 cm^-1^ difference spectra of HSA in the absence and presence of QRMAE at pH below and above two unit of their pI.

Amide I (1690–1620 cm^-1^) and amide II (1555–1535 cm^-1^) bands of IR spectra have been broadly used to characterize the chemical composition and conformational studies of proteins [47, 48]. So, we further examined the conformational alteration in amide I and II bands of HSA induced by cationic surfactant QRMAE by infrared spectroscopy. The presence of QRMAE chemical structure in the sample was confirmed by the appearance of the new broad absorption bands at 1618, 1442, 1407 and 1325 cm^-1^ and disappearance of 1755 cm^-1^ bands [32], as shown in Fig 6.

The main spectral feature of native HSA has been characterized by a strong Amide I band at 1650 cm^-1^ and an amide II absorption band at 1546 cm^-1^. The location of these bands indicates that HSA secondary structure was dominated by α-helix. The study reveals that, at pH above two units of the pI, surfactant binding produces a noticeable spectral change, compared to pH two units below pI. The spectral shifting and intensity variations of amide I band at 1650 cm^-1^ (mainly C = O stretch) [49] and other amide II band at 1546 cm^-1^ (C-N stretching coupled with N-H bending modes) [50, 51] were insignificant or absent in presence of rosin surfactants above two unit of pI, because hydrophobic contents may interfere [52] with protein hydrophilic C = O, C-N and N-H groups, as represented in Table 2. Amide I band is characteristic for α-helical contents [53] of HSA that was clearly seen in HSA-native spectra [54]. Due to aggregate formation in the presence of QRMAE, amide I band was absent, further supporting our CD results [55, 56]. Major loss of α-helical contents and a pronounce peak was found at 1618 cm^-1^ which was due to bending in primary and secondary amine group, NH_2_ bending, C = O and C = N stretching in protein.

**Table 2:** Summary of the principal infrared bands and their assignments of HSA in absence and presence of rosin surfactants.

Sample peak	Normal peak range	Functional group	Characteristics of peak
1755	1780–1720	C = O	Ester, C = O stretch [48]
1710	1725–1700	C = O	Carboxylic acid stretching
1650	1690–1620	C = O	C = O axial deformation, **Amide I** [47, 48]
1618	1640–1550	N–H	Primary and Secondary amines and Amides (bend), NH_2_bending, C = O, C = N stretching [48]
1546	1555–1535	CO–N–H	CO–N–H angular deformations, **Amide II** [47, 49]
1442	1450–1375	CH_3_	CH_3_ (bend)
1407	1410–1390	C–O	C–O stretching vibration (symmetric)
1325	1350–1000	C–N	Amines, amide III [47]

### Thermodynamic study by isothermal titration calorimetry (ITC)

ITC is routinely used to determine the thermodynamic parameters of the interaction between the ligand/micromolecules and protein/macromolecules [57, 58]. When ligands bind to protein molecules, heat is either released or absorbed, depending on the nature of ligands and proteins and it is very difficult to determine the binding process *in vivo*, whether its exothermic or endothermic [3]. Therefore, we have monitored the enthalpy alteration of protein-rosin surfactant association process *in vitro* by using ITC.

**Fig 7:**
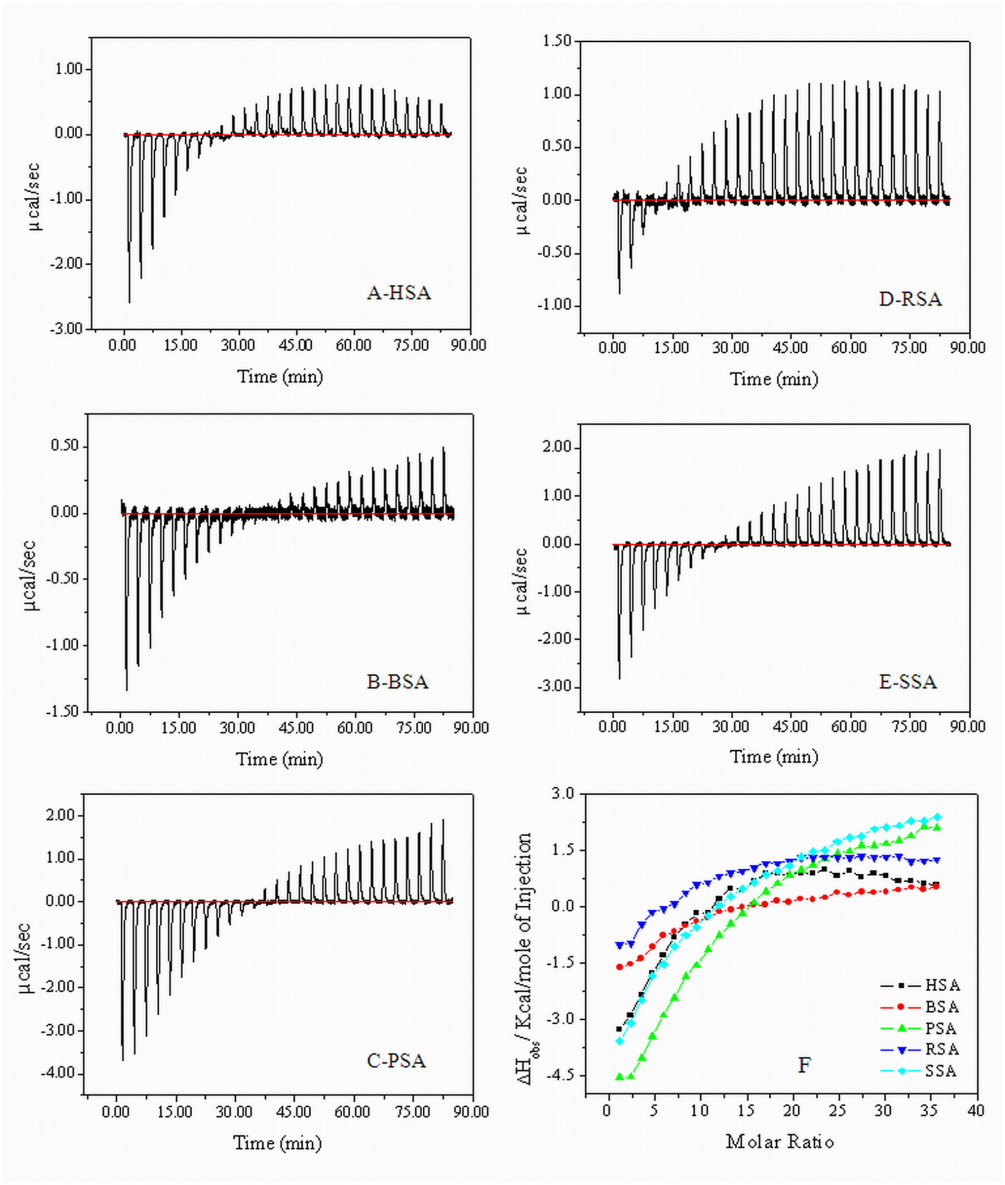
The raw data of isothermal titration calorimetry for the titration of 2500 μM QRMAE in the presence of 15 μM HSA (A), BSA (B), PSA (C), RSA (D), SSA (E) and the values of enthalpy change (F) due to the interaction of QRMAE to mammalian serum albumins at pH 7.5 and temperature 65°C. As shown in this figure firstly the interaction was exothermic that was further followed by an endothermic process that favors the aggregation process.

In the Fig 7A–7E, in the upper channel, thin lines are just a titration profile of experimental data, while the solid thick lines are fitting lines by the thermodynamic model (Fig 7F) at pH 7.5 and 65°C. It is assumed that the binding enthalpies of the surfactants become zero when the binding of surfactant saturates. Therefore, the binding enthalpies during initial process were exothermic just before the zero as shown in Fig 7F. The binding enthalpy (ΔH°) is obtained directly from the fitting progress. Initially the ΔH° values are exothermic when the alkyl chains of the cationic surfactants bind to serum albumins. As binding of surfactant molecules to serum albumins increases, which finally undergo saturation, the enthalpy values were decreased directly. After that the ΔH° values of the complex became endothermic. Bai and coworkers [59] considered that the exothermic enthalpy mainly results from electrostatic interaction between oppositely charged head-groups. Therefore, in the present study, interactions between the oppositely charged surfactants and protein that also included electrostatic binding between the head-groups, hydrogen bonds between spacer and the hydrophobic association between the alkyl chains as well as associated dehydration and counter-ion liberation of the head-groups. It has been shown that interaction between oppositely charged protein towards surfactants with long alkyl tails generally yields huge exothermic enthalpy changes [60] and the counter-ion release leads to endothermic enthalpy.

However, Norvaiaăs et al. proved that large exothermic enthalpy primarily arises from hydrophobic association of alkyl chains rather than electrostatic forces [61]. As reported by Liu *et al*., for each cationic surfactants, the head-groups have the same structures so that the electrostatic interaction between the head-groups or the hydrogen bonding between the spacers is same [60]. The variation in ΔH° value is thus mainly caused by discrepancy in hydrophobic interactions between surfactants and serum albumins [60]. ITC also gives information about the nature of interaction, whether it is hydrophobic or electrostatic [62]. In the above spectroscopic results, we found that the QRMAE only induces aggregation above two units of pI at 65°C within an hour. At pH 7.5 albumins have a net negative charge on their surface. Thus, positively charge QRMAE surfactants were bound to protein by electrostatic interaction that was an exothermic process as seen in the Fig 7F. After increasing the concentration of surfactant, the value of enthalpy gets changed and the reaction was reversed, which was followed by endothermic process that was mainly driven by hydrophobic interactions [63]. The hydrophobicity of protein was enhanced. Therefore, a hydrophobic portion of surfactant participated in the associations of protein molecules which resulted in the formation of amorphous aggregate. At room temperature (25°C) and pH 7.5, no aggregation process took place, as shown in S3 Fig [64], and there is no interferences were found with respect to the buffer used in the experiments (S4 Fig). On the other side, at pH 3.5 no interaction was found between surfactant and albumins due to electrostatic repulsion. Therefore, aggregation process was not observed (data not shown).

### Thermostability measurement by differential scanning calorimetry (DSC)

Thermal stability of albumins in absence and presence of Rosin surfactant QRMAE was monitored by DSC, which is a well-known technique to determine the stabilizing or destabilizing effect of related binding ligands on conformation of proteins. Herein, we employed DSC to investigate the effect of rosin surfactant on the thermal stability of HSA by measuring parameter related to Δ*T*_m_ which gives the information about the effect of ligands on the thermal stability of protein [58]. As shown in S5 Fig, the archetypal intemperance heat capacity curves for protein-surfactant complex in the molar ratio of 1:10 show the associated thermodynamic denaturation of albumin. Albumin-surfactant complex exhibited higher *T*_*m*_, thus showing a thermogram shift towards higher temperature (67.15°C), compared to the native albumins peaks (60.45°C) at pH 7.5. A similar, but small effect, was observed at pH 3.5 from 56.36°C to 58.15°C, as also reported previously on ligand binding protein get stabilized [29, 41]. After addition of surfactant QRMAE, positive change in heat capacity was observed, which suggested an increase in hydrophobic surface area of the protein, which is in good agreement with ITC studies. By reheating the sample after cooling just after the first run, it was observed that the thermal unfolding of albumin is irreversible process in absence and presence of surfactant. Hence to diminish the kinetic factors, slower scanning ratewas selected.

### Morphological study by scanning electron microscopy (SEM)

**Fig 8:**
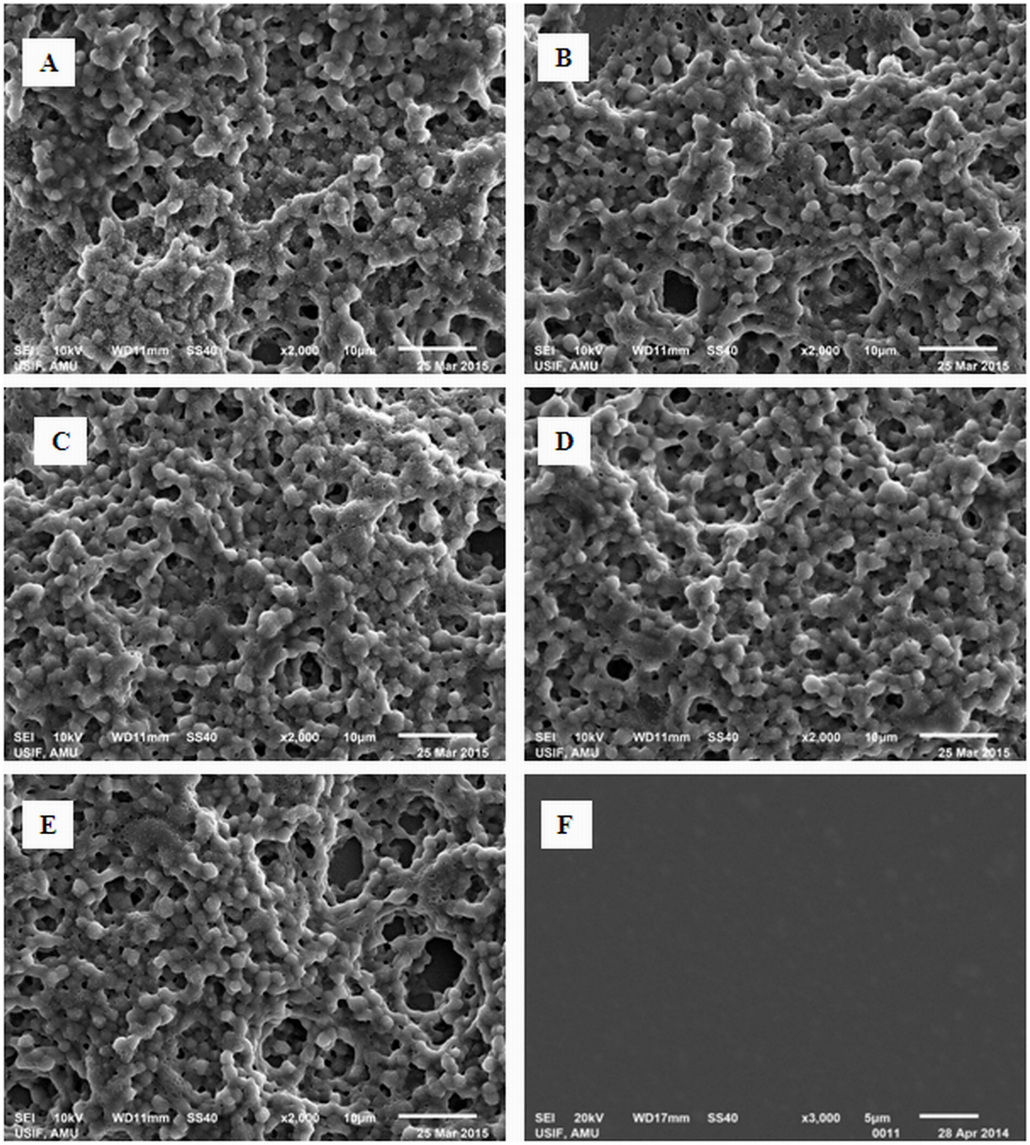
SEM images show the morphological structure of the amorphous aggregate of mammalian proteins HSA (A), BSA (B), PSA (C), RSA (D) and SSA (E) in the presence of QRMAE above two unit of their pI and HSA (F) in the presence of QRMAE below two unit of pI.

SEM is used to examine the surface morphological structure of aggregates that may be elongated or spherical which depicts the nature of the aggregation as fibrillar or amorphous. Therefore, morphology of the amorphous aggregate of albumins was further characterized by SEM. The protein samples at pH 3.5 after one hour of incubation did not show any types of aggregates in the absences and presences of QRMAE surfactant, but at pH 7.5 (two units above of albumins pI) showed aggregation only in the presence of QRMAE. The amorphous aggregates were formed are shown in Fig 8. From the SEM images our observation revealed that aggregates with several distinct diameters were formed, which merged together and formed fussiness or sponge-like structures that are known to be an amorphous aggregate of albumins. This was confirmed when QRMAE induced aggregation of albumins only at pH two units above the pI.

### Evaluating the amorphous aggregate components by fluorescence microscopy (FM)

**Fig 9:**
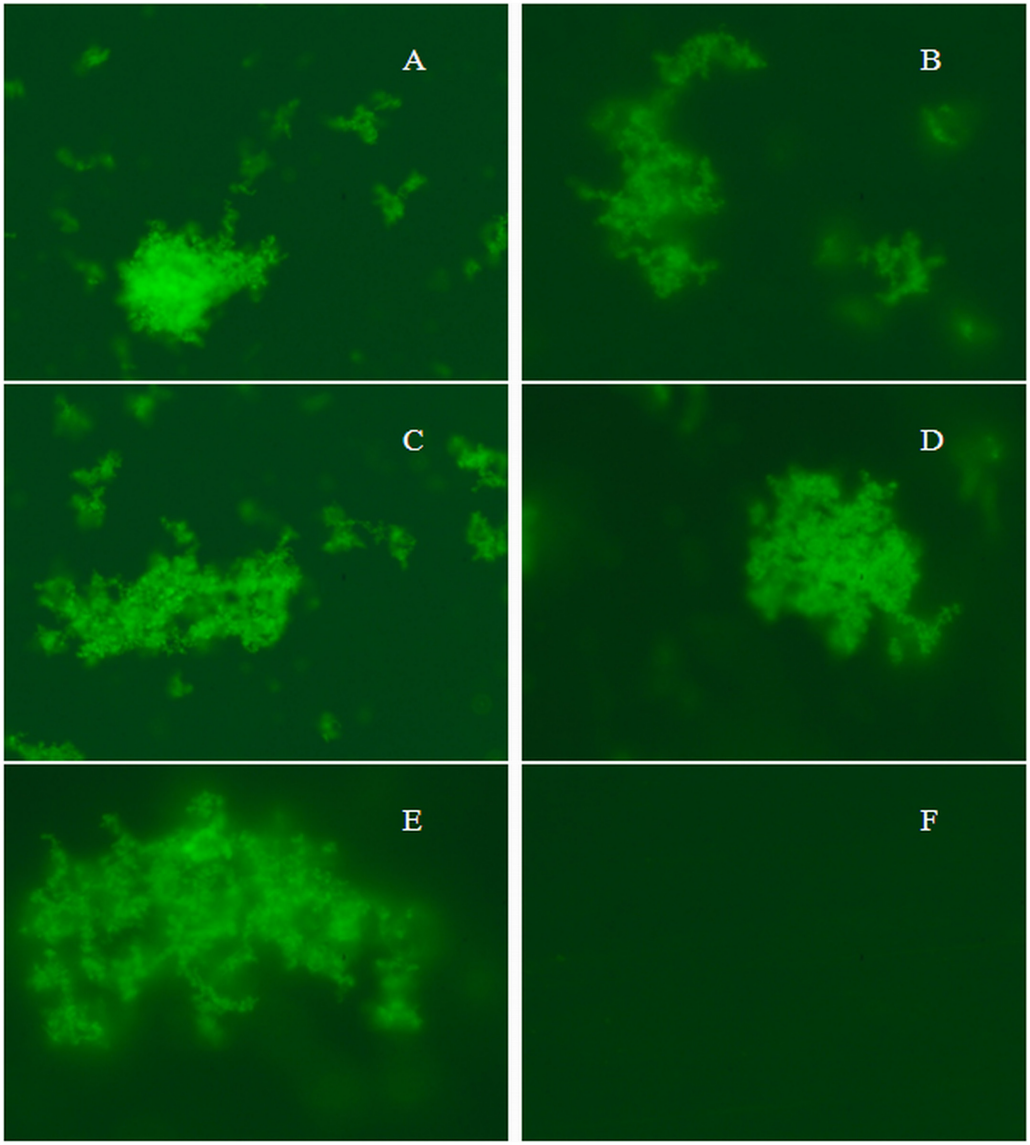
Fluorescence microscopy images of amorphous aggregate of mammalian serum albumins HSA (A), BSA (B), PSA (C), RSA (D) and SSA (E) in the presence of QRMAE above two unit of their pI and HSA (F) in the presence of QRMAE below two unit of pI.

A potentially important application of the current assay is the quantification of protein aggregate molecules co-localization. Fluorescence microscopy of aggregated protein components with ANS was co-expressed, indicating co-localization of the aggregated proteins in the sample (Fig 9). In order to confirm the nature of aggregated molecules of protein, we used ANS dye that has high propensity to bind with hydrophobic cluster of amorphous aggregate and expresses the morphological structure co-localized by fluorescence microscopy. Through fluorescence microscopic results analysis, it was further confirmed that amorphous aggregates were formed only at two units above pI and not at pH two units below protein pI, in presence of QRMAE surfactant. Therefore, it was further confirmed that rosin surfactant QRMAE induced amorphous aggregate at pH two units above albumins pI.

## Discussion

Amorphous aggregations of protein in the presence of surfactants are very widely reported in different conditions that provide a medium at which proteins occupy different conformational states and follow unfolding process. The different conditions may be high or low pH, high temperature, co-solvents and surfactant concentrations. Presently, various methodologies are used to determine the aggregation process and characterization of its morphological features, but the mechanism of *in vitro* and *in vivo* aggregation process is still unclear. The positively charged rosin modified surfactant QRMAE (similar to fatty acid molecule) has a high propensity to induce the formation of amorphous aggregate; however, the detailed study of the mechanism regarding rosin surfactants/fatty acids is still unclear. Therefore, in the present study, we have explored the pH-dependent aggregation of albumins with respect to their pI value, two units above and below their pI at which proteins have net negative and positive charges on their surfaces, respectively.

**Fig 10:**
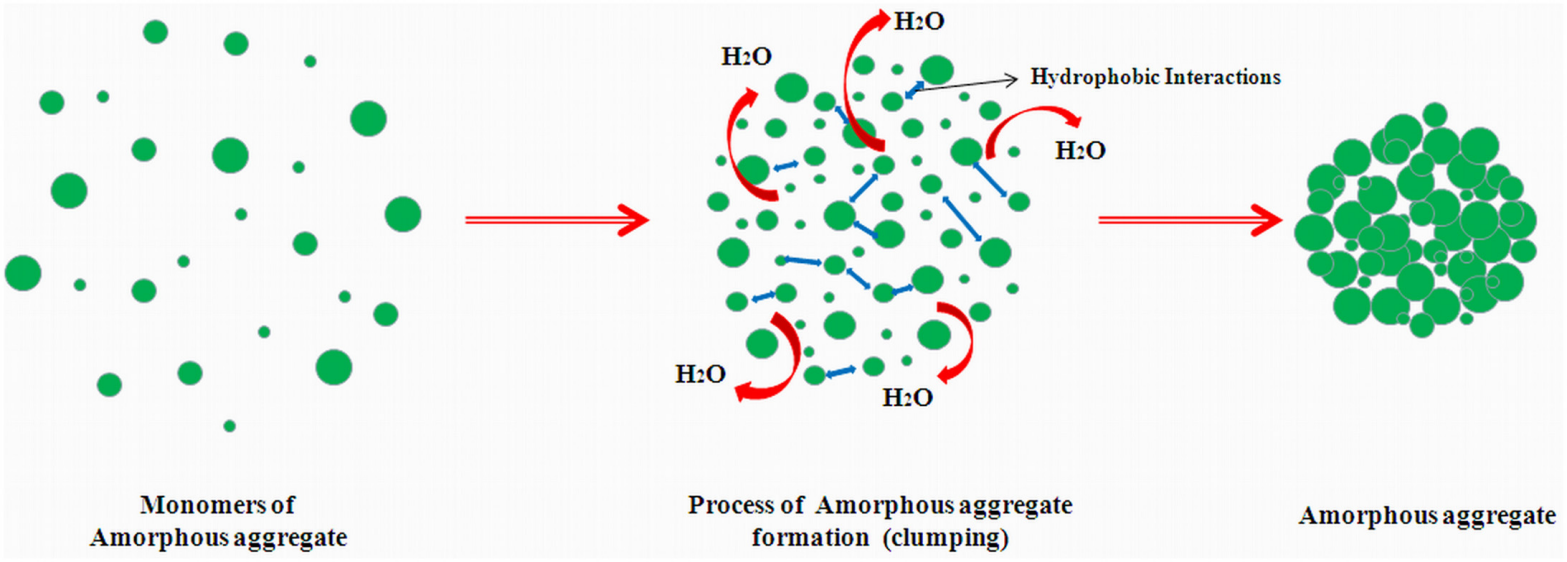
Amorphous aggregation mechanisms that follow disordered three-dimensional processes, in which monomers of aggregate can be added from any direction.

Therefore, at the time of interaction, the positive charge of surfactant neutralized the negative charge of the protein resulting in the formation of amorphous aggregate. According to this study, electrostatic interactions play a very significant role in determining the rosin surfactant induced amorphous aggregation. Amorphous aggregation can be considered as a disordered three-dimensional process, where monomers can be added from any direction as seen in Fig 10 and form a large amorphous cluster by hydrophobic interactions in which the water molecules present inside or nearby were expelled out from the cluster. According to amorphous aggregation model of Strank *et al*. [8], aggregate mass *M*(*t*) will increase with time as monomers keep on adding and forming clusters, and the increment rate will be depend on the concentration, *C*, of available monomers, that rate will follow a power law of the form and will be represented as:

dMdtαCr (3)

for the same as yet undetermined exponent, r ≥ 0, that is used in general and the deviated from a classical value of 1 for first order kinetics.

In present work, we firstly optimized the surfactant concentration for maximum aggregation, and then incubated the mammalian serum albumins (5 μM) with cationic rosin surfactant QRMAE at pH above two units of their pI at 65°C for 60 min. For choosing maximum aggregation point we performed surfactant concentration dependent turbidity and RLS experiments at 350 nm to obtain different concentrations of QRMAE at which aggregation was observed and it was found that at ~50-fold molar ratio higher than the protein concentration, maximum aggregation point was achieved, as seen in Figs 1A and 2A.

Further, all the experiments were performed at that particular concentration of the surfactant. To observe the turbidity and RLS values at 350 nm, the experiments were performed at pH above two unit of pI in the presence of QRMAE and the obtained values were higher as compared to below two unit of pI, as shown in Figs 1B and 2B. The ANS emission spectra are related to the presence of hydrophobic clusters, indicating the formation of new hydrophobic cluster during the aggregation process. We observed a pronounced ANS fluorescence intensity of the sample above two unit of pI due to the exposure of hydrophobic surface [65], ANS is a probe that binds to hydrophobic clusters already present in the protein structure or eventually formed during the evolution of the aggregation process [38]. The behavior of ANS emission indicates the formation of amorphous aggregates (Fig 3).

In order to confirm the nature of aggregates ie; amorphous vs. fibrillar, we performed ThT dye binding assay that mainly binds to amyloid or fibrillar structure of protein. ThT is known to bind poorly to amorphous aggregate and thus shows negligible change in fluorescence intensity as shown in Fig 4. Basically, ThT binds to cross β-sheet structure of fibrillar protein but in case of amorphous aggregation ThT does not bind due to the absence of cross β-sheets structure. We found similar ThT binding patterns for all the proteins and this observation was further supported by CD results (Table 1). According to FT-IR/ATR study, amide I (1690–1620 cm^-1^) reflects almost pure vibrational character, since it consists mainly of carbonyl stretching vibration mode of the peptide bond. In HSA incubated with QRMAE above two unit of pI, amide I and amide II bands were insignificant as compared to both native HSA (pH 7.5 and 3.5) and HSA complex with QRMAE at below two unit of pI. Amide I band is more sensitive than the amide band II regarding protein secondary structure [48]. These results clearly indicate the loss of α-helical structure of protein. The drastic reduction was observed in α-helix conformation and the significant enhancement in random coil and β-sheet was consistent with the CD results which also showed the reduction of α-helix and an increase of random coil and turn structure due to protein unfolding process that finally leads to the formation of amorphous aggregates.

We further explored aggregation behavior of albumins induced by QRMAE by using ITC to distinguish the nature of interactions between surfactants and protein molecules at 65°C. ITC results revealed that the interaction of surfactants to albumins involved great enthalpy alterations. The exothermic and endothermic enthalpy changes indicated that firstly electrostatic interactions were involvement which induced a conformational alteration in protein, followed by hydrophobic interactions which were responsible for the aggregation of proteins. The morphological features of aggregate were also characterized by SEM and electron microscopy that provided the topological information about aggregated molecules and confirmed the nature of aggregated protein as amorphous. According to DSC analysis, after interaction of surfactant with albumins, thermal stability of overall complex was increased and the aggregation process was started at pH 7.5, but not at pH 3.5, as suggested by enthalpy alteration profile by DSC.

Aggregation occurs enthusiastically in all mammalian proteins most probably due to the neutralization of negative charges (above pI) as well as hydrophobic interaction between QRMAE and albumins. However, to further investigate the main cause of initiation of aggregation process we performed the same experiment at pH below two unit of their pI in the absence and presence of QRMAE. Unfortunately, we did not find any aggregates under these circumstances due to the presence of positive charge on protein surface (below pI) which were involved in electrostatic repulsion i.e. charge-charge repulsion instead of electrostatic interaction between protein-surfactants. Similar finding was also reported by our group previously but the case were just apposite [7], where the SDS (with negative charge) induced amyloid formation at pH below pI of proteins.

**Fig 11:**
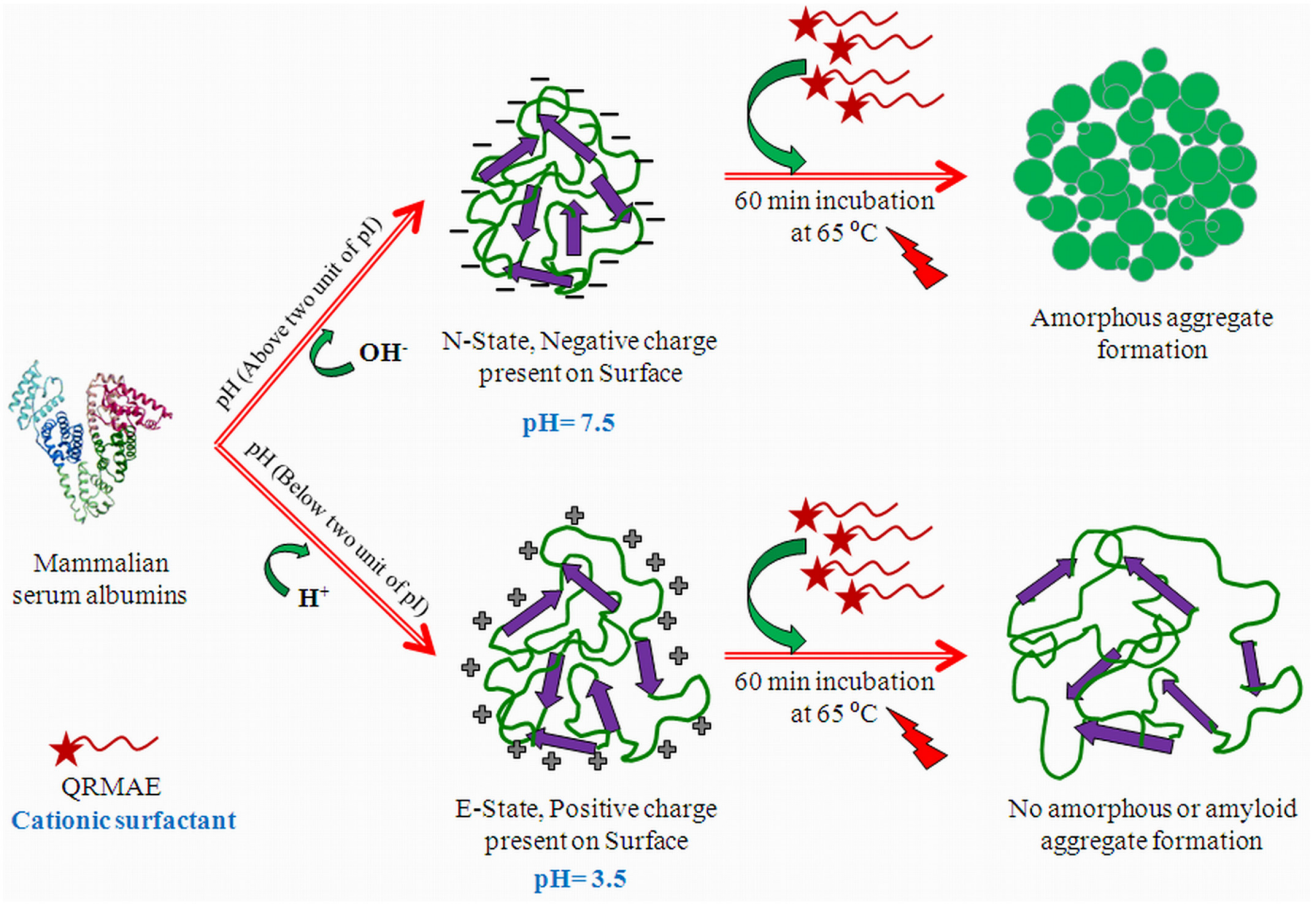
The hypothetical schematic presentation of QRMAE induced pH dependent amorphous aggregation of mammalian serum albumins.

Together, these findings suggested that the electrostatic interactions play an important role in the field of aggregation followed by hydrophobic interaction. However, we cannot ignore the contribution of 17-carbon long chain of QRMAE in hydrophobic interaction which may lead to the accumulation and, finally, the formation of amorphous aggregate clusters. Fig 11 shows a hypothetical model representing the major findings of this study, where mammalian serum albumins were incubated with positively charged QMARE at pH two units above pI for 60 min at 65°C. The net negative charge on the surface of albumin was neutralized by the positive charge of QMARE through electrostatic interaction. This paves a favorable condition for hydrophobic interactions to induce the formation of amorphous aggregates. The non-polar hydrocarbon chain of QRMAE and the hydrophobic patches present in albumin (mainly in sub-domain IIA and IIIA corresponding to Sudlow site I and Sudlow site II) [26, 66] play an important role in the formation of amorphous aggregates. On other hand, the proteins at pH below two unit of pI under the same conditions, possessed net positive charge and thus did not show any types of aggregation, mainly due to charge-charge repulsion.

## Conclusion

From the above results, we can conclude that the positively charged rosin modified surfactant QRMAE has great potential to induce amorphous aggregate formation under particular conditions *i*.*e*.; at high temperature (65°C) for a specific time (~60 min) at pH above two unit of protein pI. We have found that initially the electrostatic bond plays an important role in protein-surfactant interaction, followed by hydrophobic interaction which is responsible for the collapse of hydrophobic patches present in protein and surfactant that facilitates the formation of clusters of amorphous aggregates. But, this hydrophobic interaction alone is unable to form aggregates without electrostatic interactions that responsible to initiate the aggregation process but not as seen in the case pH below two unit of the protein pI where aggregation process was resisting.

## Supporting Information

S1 Fig Schematic representation of synthesis of nobel rosin compound quaternary amine of rosin diethylaminoethyl ester (QRMAE) from original/parental compound abietic acid. (TIF)

S2 Fig Near- CD Spectra for tertiory structure of albumin. Near- CD Spectra of HSA in the presence and absence of rosin surfactant QRMAE at pH below two unit of pI (pH 3.5) and pH above two unit of pI (pH 7.5).
(TIF)

S3 Fig ITC raw data at room temp. ITC raw data for the titration of rosin surfactant QRMAE (500 μM) with HSA (15 μM) at pH 7.4 and 25°C, showing the calorimetric response as successive injections of surfactants are added to the sample cell. Integrated heat profiles of the calorimetric titration are shown in the lower panel.
(TIF)

S4 Fig ITC control ITC raw data for the titration of rosin surfactant QRMAE (500 μM) with 20 mM buffer at pH 7.4 and 25°C, showing the calorimetric response as successive injections of surfactants are added to the sample cell. Integrated heat profiles of the calorimetric titration are shown in the lower panel.
(TIF)

S5 Fig DSC thermogram DSC profile of HSA in the presence and absence of rosin surfactant QRMAE at pH below two unit of pI (pH 3.5) and pH above two unit of pI (pH 7.5).
(TIF)
