# Supplementary figures and images for: Rosin Surfactant QRMAE Can Be Utilized as an Amorphous Aggregate Inducer: A Case Study of Mammalian Serum Albumin
Source: PLoS One. 2015 Sep 29;10(9):e0139027. doi: 10.1371/journal.pone.0139027 (PMC4587963; doi:10.1371/journal.pone.0139027)

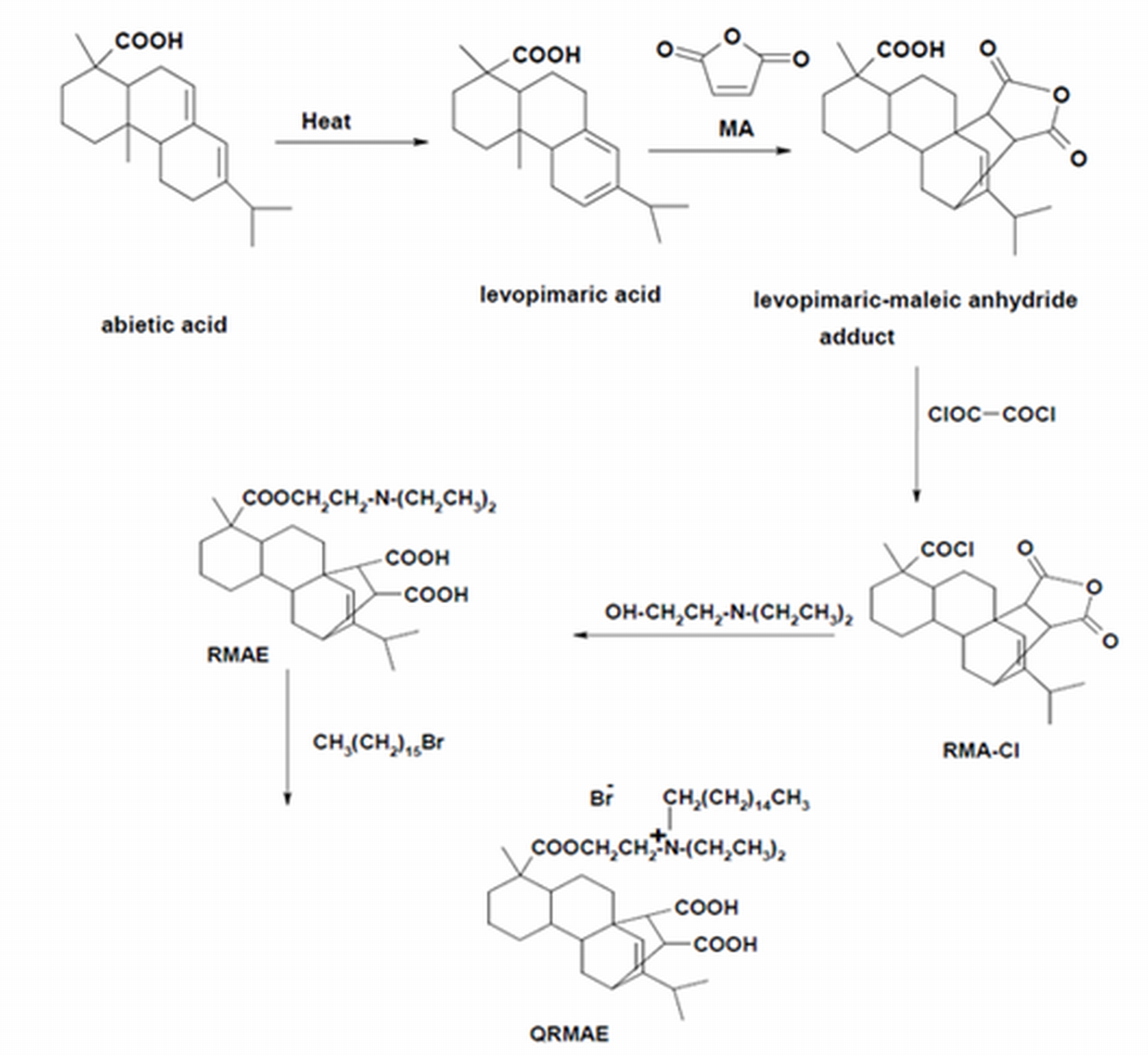

Supplement: S1 Fig — (TIF) [file pone.0139027.s001.tif]

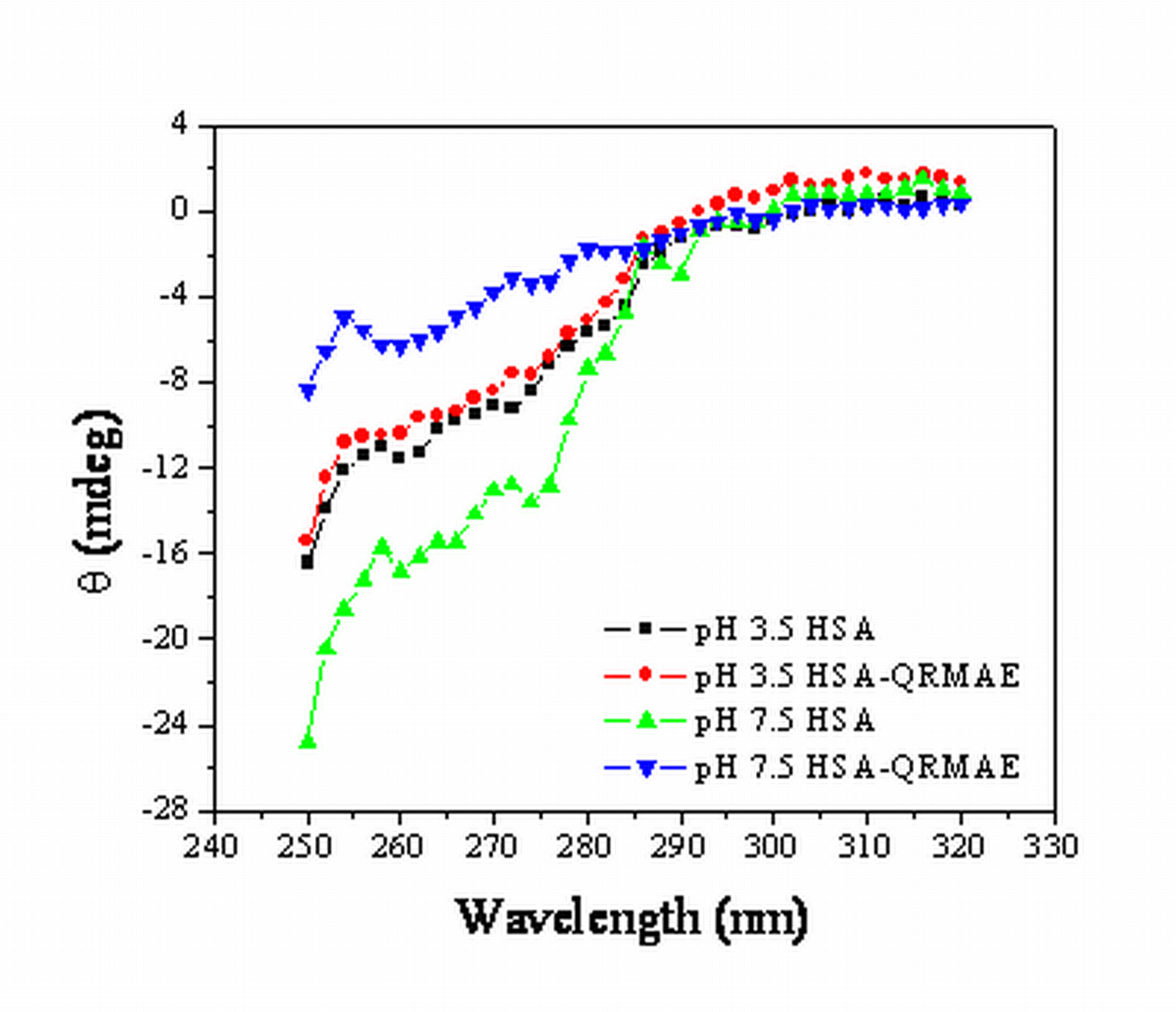

Supplement: S2 Fig — Near- CD Spectra of HSA in the presence and absence of rosin surfactant QRMAE at pH below two unit of pI (pH 3.5) and pH above two unit of pI (pH 7.5). (TIF) [file pone.0139027.s002.tif]

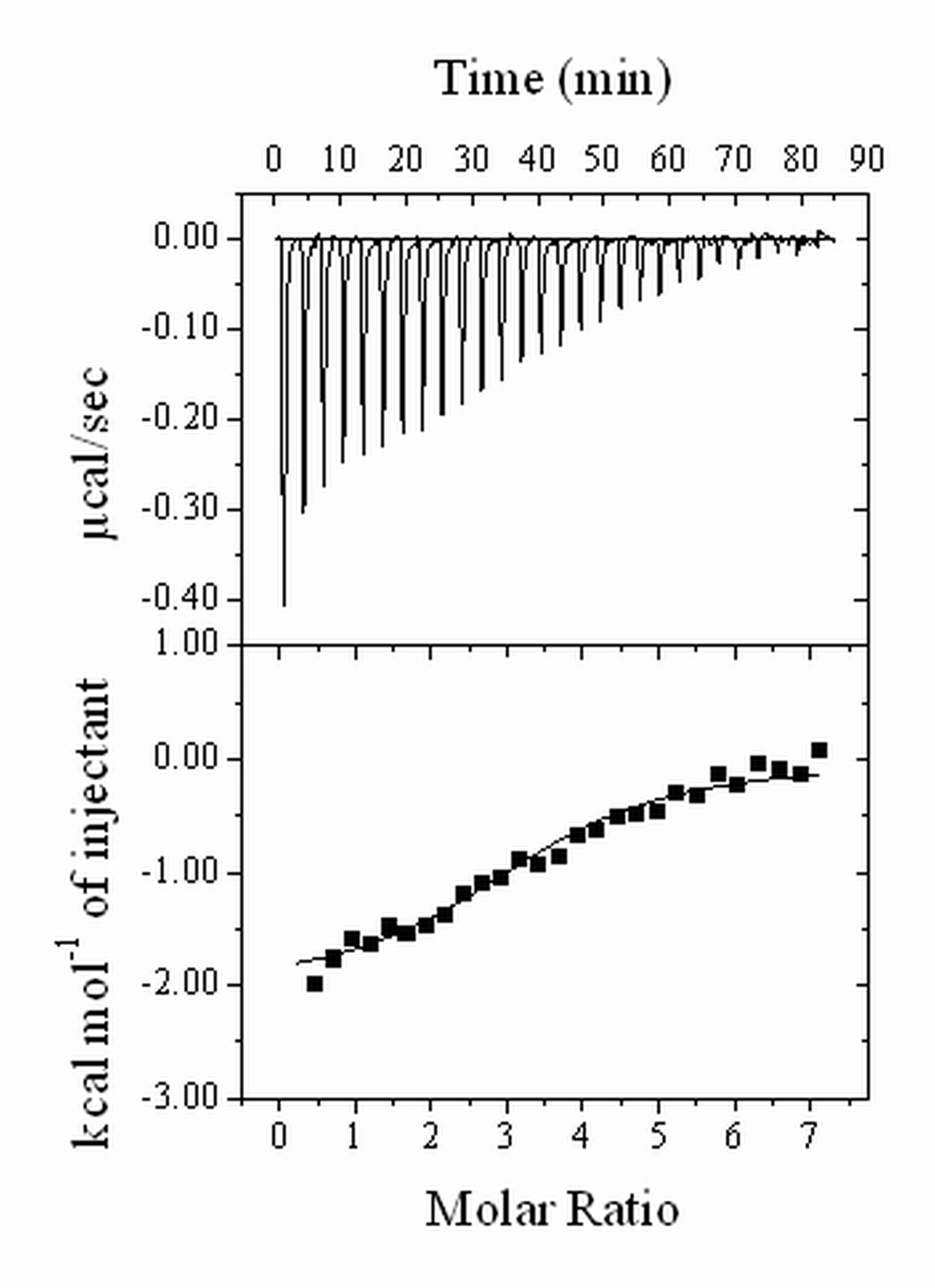

Supplement: S3 Fig — ITC raw data for the titration of rosin surfactant QRMAE (500 μM) with HSA (15 μM) at pH 7.4 and 25°C, showing the calorimetric response as successive injections of surfactants are added to the sample cell. Integrated heat profiles of the calorimetric titration are shown in the lower panel. (TIF) [file pone.0139027.s003.tif]

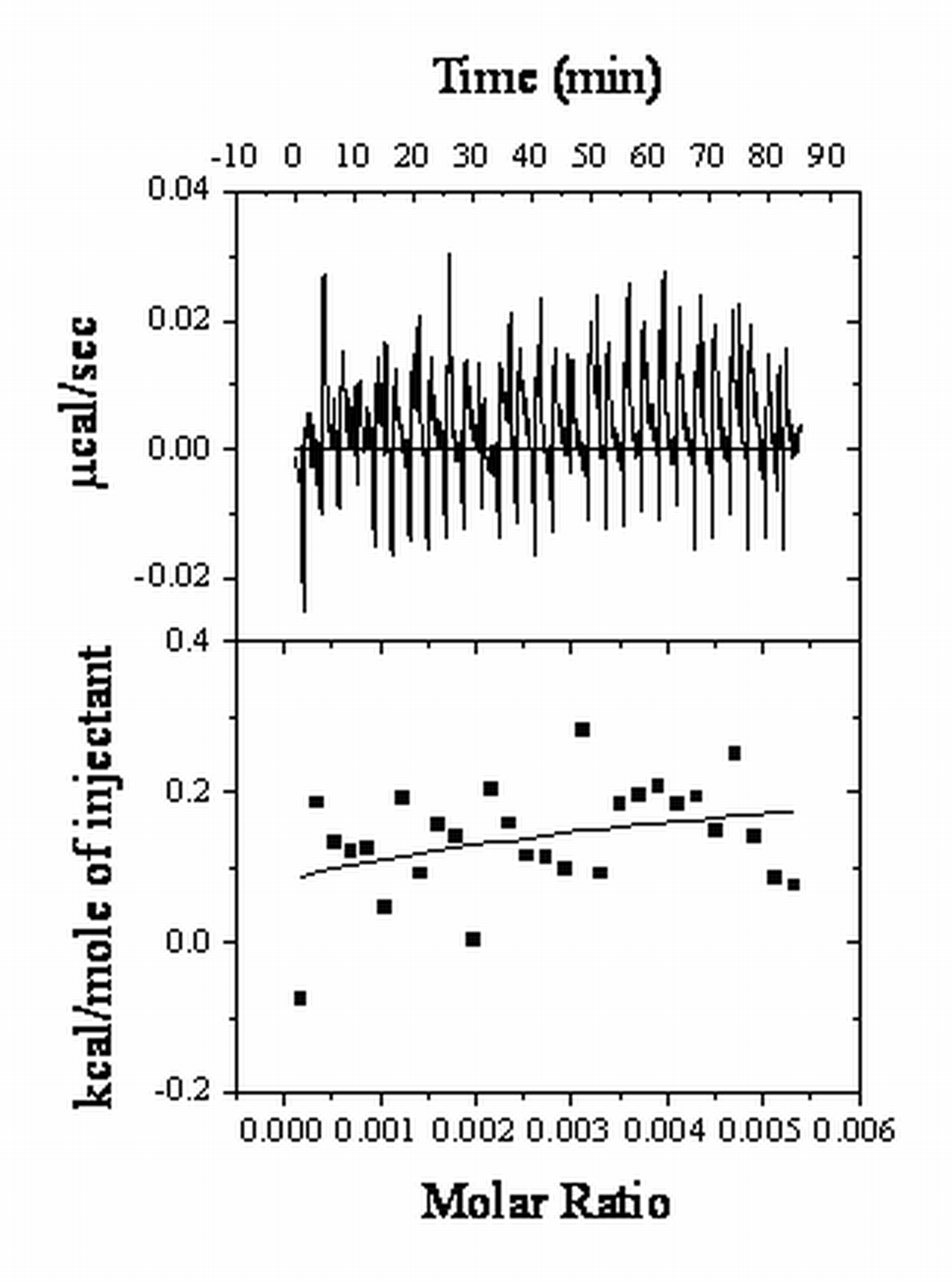

Supplement: S4 Fig — ITC raw data for the titration of rosin surfactant QRMAE (500 μM) with 20 mM buffer at pH 7.4 and 25°C, showing the calorimetric response as successive injections of surfactants are added to the sample cell. Integrated heat profiles of the calorimetric titration are shown in the lower panel. (TIF) [file pone.0139027.s004.tif]

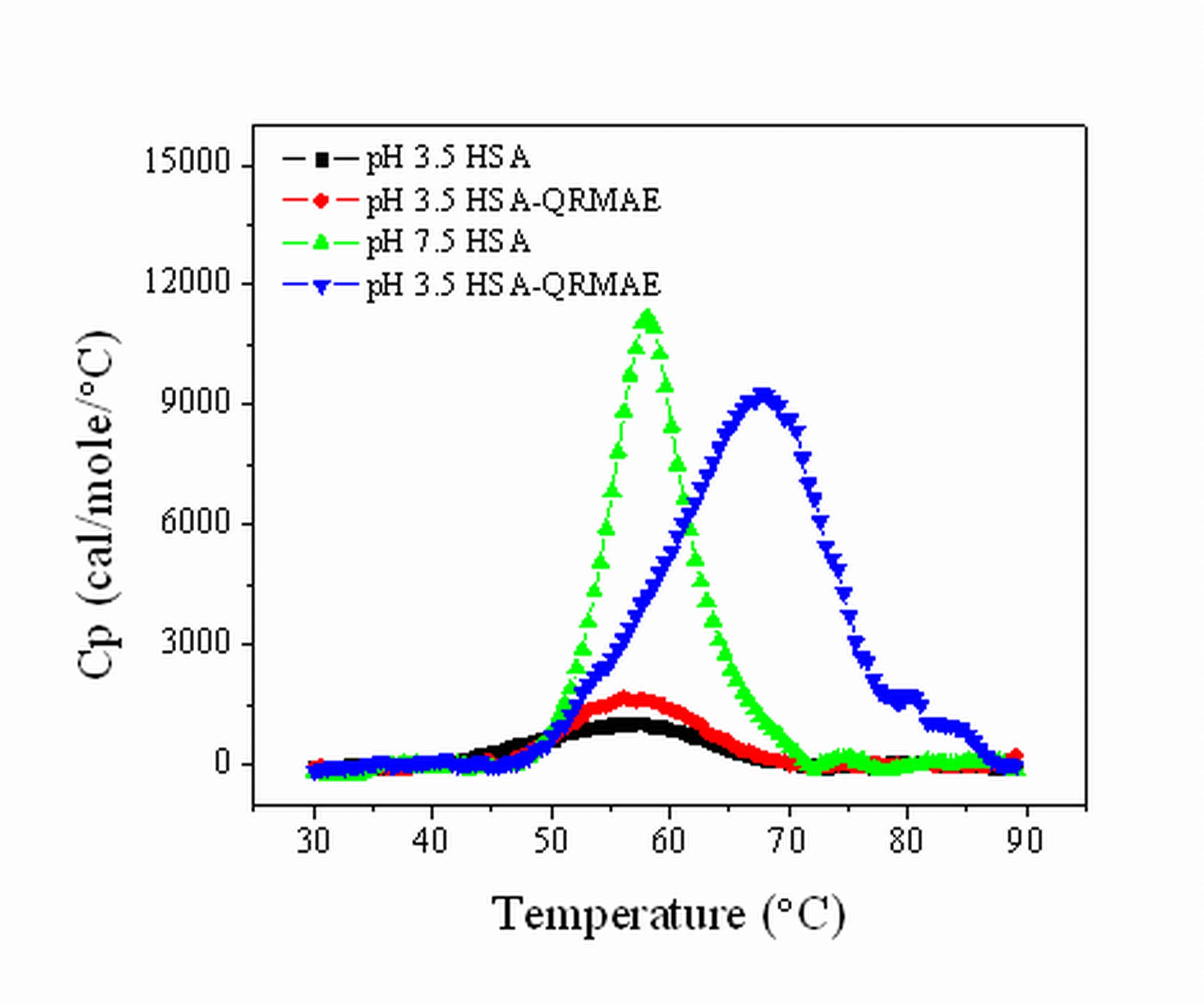

Supplement: S5 Fig — DSC profile of HSA in the presence and absence of rosin surfactant QRMAE at pH below two unit of pI (pH 3.5) and pH above two unit of pI (pH 7.5). (TIF) [file pone.0139027.s005.tif]
